# Longitudinal SARS-CoV-2 seroprevalence among learners, parents, and teachers in a South African school community

**DOI:** 10.3389/fpubh.2026.1770703

**Published:** 2026-06-19

**Authors:** Reshmi Dassaye, Terusha Chetty, Brodie Daniels, Trisha Ramraj, Samkelisiwe Buthelezi, Khanya Mohlabi, Tarylee Reddy, Isaac Singini, Nomonde Gwebushe, Ameena Goga

**Affiliations:** 1 HIV and Other Infectious Diseases Research Unit, South African Medical Research Council, Cape Town, South Africa; 2 Discipline of Public Health Medicine, University of KwaZulu-Natal, Durban, South Africa; 3 Biostatistics Research Unit, South African Medical Research Council, Cape Town, South Africa; 4 Department of Paediatrics and Child Health, University of Pretoria, Pretoria, South Africa

**Keywords:** COVID-19, SARS-CoV-2, seroprevalence, learners, longitudinal assessment

## Abstract

**Introduction:**

Longitudinal assessments of SARS-CoV-2 seroprevalence among a triad of parents, teachers and children in low-middle income countries are scarce. CoKiDSS assessed changes in seroprevalence among asymptomatic primary school learners, their parents, and teachers in a semi-rural South African school setting.

**Methods:**

SARS-CoV-2 seroprevalence was estimated at baseline (May–August 2023) and follow-up (October 2023) using a point-of-care COVID-19 IgG/IgM test. Results were adjusted for test sensitivity and specificity. Between-group differences and within-participant serostatus changes were assessed using Fisher’s exact test, and McNemar’s test, respectively.

**Results:**

292 participants (203 learners, 71 parents, and 18 teachers) were included in this analysis. At baseline, overall (all group combined), SARS-CoV-2 IgG seroprevalence was 73% (unadjusted) and 78% (adjusted for test sensitivity). Seropositivity was higher among learners compared to parents (74% unadjusted to 77% adjusted vs. 66% unadjusted to 69% adjusted), with teachers showing the highest levels (94% unadjusted to 99% adjusted). Approximately 2.7% of learners were IgM seropositive, while about 25% of participants were IgG/IgM seronegative (24% of learners, 34% of parents, and 5.6% of teachers). At follow-up, overall SARS-CoV-2 IgG seroprevalence rose to 85% (unadjusted) to 95% (adjusted), with increases across all groups (learners: 96%, parents: 90%, teachers: 100%). A group-level longitudinal assessment showed there were significant increases in IgG seropositivity among learners (74–90%, *p* < 0.0001) and parents (66–85%, *p* = 0.015), while teachers remained consistently seropositive. The individual-level longitudinal assessment demonstrated that 69% of participants remained seropositive at follow-up, while 20% of those seronegative at baseline were seropositive at follow-up (*p* < 0.001).

**Discussion:**

Three years after the COVID-19 pandemic, despite no reported symptoms, and no vaccination campaigns, SARS-CoV-2 IgG seroprevalence increased over the 3–4-month follow-up period, particularly among unvaccinated learners, suggesting ongoing exposure and antibody persistence, although causal attribution cannot be established. Teachers maintained consistently high seroprevalence, likely reflecting sustained antibodies following prior infection or vaccination. Data suggest high levels of circulating virus and asymptomatic spread created endemicity in this environment. These findings should not guide vaccination policy but instead inform understanding of population-level seroprevalence.

## 1 Introduction

The coronavirus disease 2019 (COVID-19) pandemic caused by severe acute respiratory syndrome coronavirus 2 (SARS-CoV-2) and its variants led to more than 770 million infections and over 7 million deaths globally (1). South Africa reported about 4 million cases of SARS-CoV-2 infection with over 100,000 deaths (1). Early in the pandemic, children’s role in SARS-CoV-2 transmission remained unclear, even though children are well-documented key transmitters of other respiratory viruses such as influenza (2). Consequently, many countries including South Africa adopted non-pharmacological interventions (NPIs), such as school closures, to reduce virus transmission, curb community spread and prevent overwhelming the healthcare system (3). Globally, schools were fully closed for approximately 20 weeks and partially closed for an additional 22 weeks between March 2020 and July 2022 (4). The South African government closed schools on 18th March 2020, and implemented a phased staggered, partial reopening between June and August 2020 (5). A second round of school closures occurred in 2021, with daily school attendance officially resuming on 2 August 2021. The direct impact of school closures on children and adolescents was extensive, ranging from disruption in education and learning to affecting their physical and mental well-being, as well as increasing their vulnerability to child labor, gender-based violence, early marriages, early pregnancies and other risks (6, 7). SARS-CoV-2 infections in children varied from asymptomatic or mild-to-moderate acute illness to post-acute complications, including multisystem inflammatory syndrome in children (MIS-C) and long COVID. The varied clinical presentation of SARS-CoV-2 infection among children and adults suggests that age may govern the host response to SARS-CoV-2, as evident for other viral infections (8–14).

From 2020 to 2022, South Africa went through multiple COVID-19 waves, each dominated by different variants including Alpha, Beta, Delta, and Omicron (BA.1/BA.2). Across the waves, children and adolescents demonstrated altered epidemiology, with the majority of cases being asymptomatic (15). In many countries, including South Africa, children were not prioritized in the initial COVID-19 vaccination programs due to their low susceptibility to severe illness, concerns about vaccine safety, and parental hesitancy (16, 17). Several COVID-19 school-based studies in developed countries investigated SARS-CoV-2 diagnosis and subsequent contact tracing among symptomatic participants during the pandemic (18–26). However, children and adolescents with asymptomatic or mild COVID-19 may have been undetected, and their role in COVID-19 transmission within schools remains elusive. SARS-CoV-2 seroprevalence studies in school settings are advantageous for several reasons, including accurately estimating infection rates, understanding transmission patterns, assessing population immunity, guiding public health policies, identifying high-risk groups, evaluating the effectiveness of preventive measures, and monitoring immunity over time (27, 28). Three community-based studies conducted in South Africa during the Beta, Delta, and early Omicron waves indicate that overall seroprevalence increased with each wave (29–31). Further, adolescents exhibited rates similar to adults, while younger children had lower seroprevalence levels. Given the dearth of surveillance among school-going children in low-middle income settings, we conducted the COVID Kids School Study (CoKiDSS). Our cross-sectional survey, described high, comparable SARS-CoV-2 IgG seroprevalence among primary school learners in grades 1–7, their parents and teachers in semi-rural Ndwedwe, KwaZulu-Natal, South Africa between May 2023 and August 2023 (32). Here, we present findings from the CoKiDSS follow-up survey conducted in October 2023, which aimed to assess longitudinal changes in SARS-CoV-2 seroprevalence in a subset of CoKiDSS participants.

## 2 Methods

### 2.1 Study setting, population and design

The protocol of this follow-up survey is reported elsewhere (33). The follow-up survey was part of the CoKiDSS, a pilot study, implemented between May 2023 and October 2023 in the under-served, semi-rural, Ndwedwe local municipality of the iLembe District, KwaZulu-Natal, South Africa. The province of KwaZulu-Natal is characterised by a high burden of HIV and tuberculosis (TB), with approximately 1.98 million people living with HIV and persistently high TB incidence, including substantial HIV/TB co-infection (34, 35). Ndwedwe has ~ 165, 826 residents, mainly of black ethnicity and are predominately engaged in subsistence farming (36). Schools in South Africa were closed in early March 2020 and fully reopened in August 2021 under NPIs which were progressively adjusted as the number of COVID-19 cases in the community dissipated and the level of the national lockdown was reduced and eventually ended. At the time of the survey, the total number of actively tested cases of COVID-19 in South Africa was 61,362 (37) and the use of NPIs in schools waned. South Africa commenced the roll-out of COVID-19 vaccinations on 17 February 2021, initially prioritizing healthcare workers, frontline workers and individuals ≥ 60 years of age. As of 27 March 2023, 38,717,957 COVID-19 vaccines were administered in South Africa (38). South African children aged 5–12 years of age were only eligible for a COVID-19 vaccination in March 2023 if they were at risk of severe COVID-19 due to underlying comorbidities (poorly controlled asthma, chronic heart, kidney, liver, neurological or digestive system conditions, endocrine disorders including diabetes, immune suppression, asplenia or a dysfunctional spleen or a serious genetic defect). For this study, a convenient sample of two public primary schools that were large enough to contribute to the target sample size was selected (33). The first 300 learners in grades 1–7 attending the first primary school, their parents and teachers that participated in the cross-sectional survey (aka baseline survey) were invited to participate in the follow-up survey. The surveys were conducted approximately 3–4 months apart during the follow-up period, there were no vaccination or immune boosting campaigns, and limited vaccination services for both adults and children. Most vaccination and immune boosting, in this setting, occurred during 2021-mid-2022, when these services were available for adults only.

### 2.2 Timeline of surveys and data collection

The baseline and follow-up surveys were implemented between May 2023–August 2023 and October 2023, respectively. At each survey, the enrolled learner, parent and teacher completed an interviewer- or self-administered questionnaire that was captured on a Research Electronic Data Capture (REDCap) (39, 40) database and a fingerprick of blood was collected for SARS-CoV-2 antibody testing. In both surveys, self-reported data were collected on sociodemographic and basic health information; COVID-19 history; symptoms of long COVID and COVID-19 vaccination status of learners, parents and teachers approximately 3 years into the pandemic.

### 2.3 Serological testing

A South African Health Products Regulatory Authority (SAHPRA)- approved point-of-care (POC) COVID-19 antibody test, the COVID-19 IgG/IgM Rapid Test Cassette from Orient Gene Biotech, Zhejiang, China, was used to qualitatively detect IgG and IgM antibodies directed against recombinant SARS-CoV-2 antigens from finger-prick blood samples. The assay detected antibodies directed against both the spike and nucleocapsid proteins of SARS-CoV-2 and thus could not differentiate antibodies arising from natural infection or those induced by vaccination. The POC test is a lateral flow immunochromatographic assay with a reported relative sensitivity of 96.2% and specificity of 100% according to the manufacturer’s package insert. The use of a POC lateral flow assay enabled rapid, minimally invasive serological testing and was considered appropriate for repeated testing in a school-based field setting involving both children and adults. IgG and IgM seropositivity were determined by the presence of separate visual bands and interpreted as per the manufacturer’s instructions. Test results were read independently by trained study personnel to ensure consistency in interpretation. All staff performing the POC test were trained using a standardised operating procedure. The same staff conducted and interpreted all tests, and quality control was performed through weekly spot checks by an experienced study coordinator.

The results from the baseline and follow-up surveys for the 292 participants with repeated SARS-CoV-2 antibody testing were combined for longitudinal analysis of serological outcomes.

### 2.4 Statistical analysis

Participant characteristics at baseline and follow-up were summarized using descriptive statistics. Categorical variables were presented as frequencies and percentages. The primary objective was to evaluate changes in SARS-CoV-2 seroprevalence between baseline and follow-up surveys. Unique participant identifiers enabled longitudinal tracking of serological status over time.

To account for imperfect diagnostic accuracy, adjusted prevalence estimates were calculated using the Rogan Gladen estimator; assuming a point-of-care (POC) test sensitivity of 96.2 and 100% specificity based on manufacturer validation; however, real-world specificity may differ from manufacturer-reported estimates. This method corrects observed prevalence for misclassification arising from imperfect sensitivity and specificity and is widely used in seroepidemiological studies. Crude and sensitivity-adjusted seroprevalence estimates were generated at each survey round and stratified by participant group (learners, parents, teachers). Negative and indeterminate results were not adjusted, as such corrections are not methodologically appropriate. Group-level comparisons of results were assessed using Chi-square of Fisher’s exact test, where appropriate.

Paired analyses were conducted among participants with valid results in both surveys. Within-participant changes in antibody status (IgG+, IgM+) were evaluated using a McNemar’s test and exact McNemar (for small counts), which assesses whether the proportion of participants changing from positive to negative (or vice versa) differs significantly over time. A significant *p*-value (<0.05) indicates a non-random shift in serostatus between surveys. Missing data was handled using complete-case analysis. Sensitivity analyses were conducted to evaluate the robustness of our findings under alternative assumptions regarding missingness. We conducted a complete-case analysis as the primary comparison. To assess the potential influence of missing outcome data, we recomputed seroprevalence estimates under a ‘best-case’ scenario (all missing outcomes treated as seronegative) and a ‘worst-case’ scenario (all missing outcomes treated as seropositive). These analyses were used to evaluate the degree to which missingness could shift the observed estimates. All statistical analyses were performed using R (version 4.0.3) and Stata (version 16). In R, we used stats for McNemar’s tests, dplyr for data manipulation, and flextable and officer for creating and exporting summary tables to Word. Seroprevalence estimates with 95% confidence intervals were computed using prop.test() from base R. In Stata, we conducted analogous analyses using mcc, tabi, among others. This detailed description ensures full reproducibility.

## 3 Results

### 3.1 Characteristics of the participants (i.e., learners in grades 1–7, their parents and teachers) at the follow-up survey

**Figure 1:**
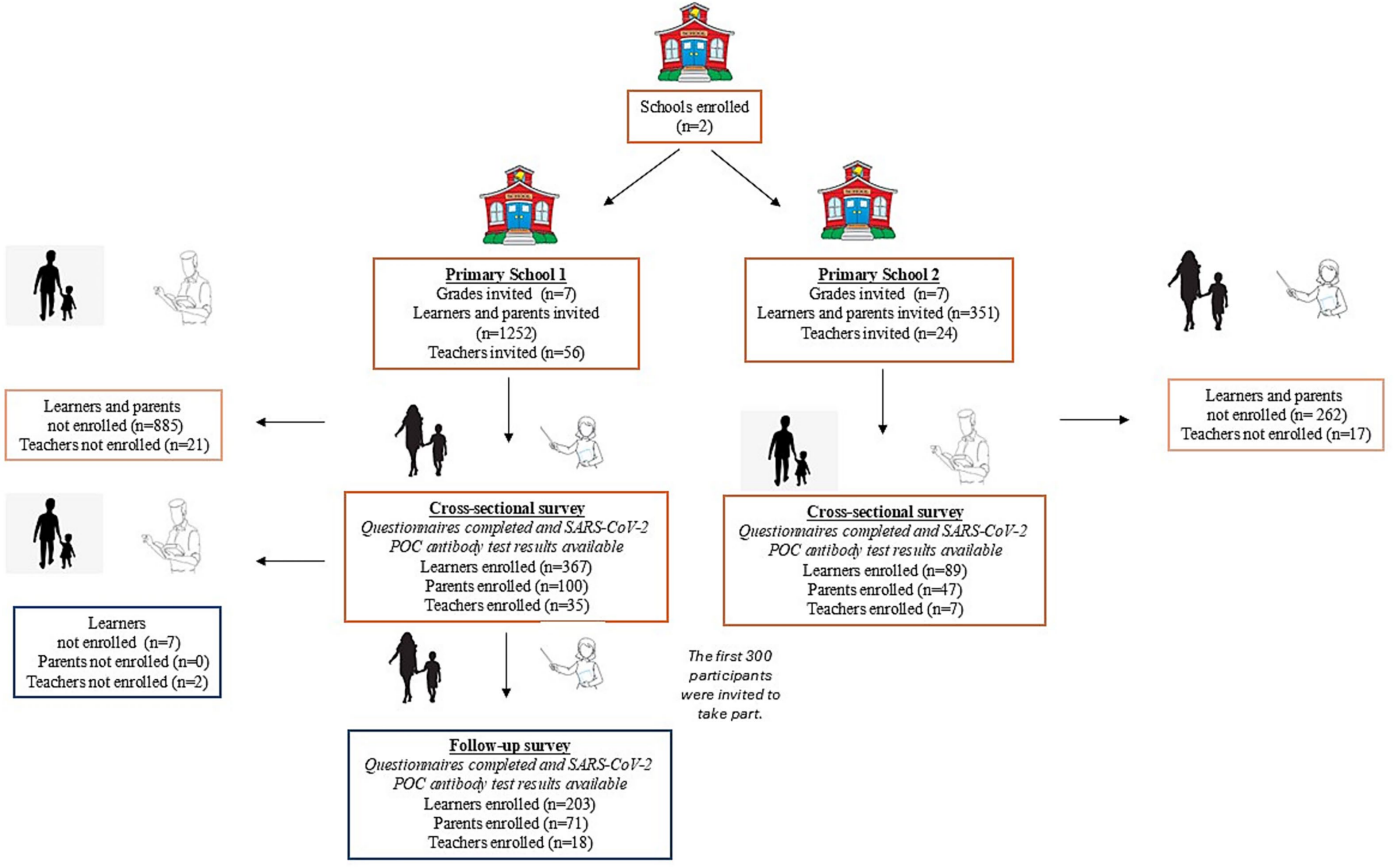
Flow chart of participants enrolled in the CoKiDSS – follow-up survey.

In total, 292 participants (203 learners, 71 parents and 18 teachers) were followed up in October 2023 (Table 1). Figure 1 shows the flow of 292 enrolled participants with available questionnaire information and serological test results. In the follow-up survey, the participation among learners in senior primary (62.1%) was greater than junior primary (37.9%). Participation was nearly evenly distributed between male and female learners, with 95 male learners (46.8%) and 108 female learners (53.2%). However, most parents and teachers who participated in the follow-up survey were female (parents *n* = 62, 87.3%, and teachers *n* = 16, 88.9%, respectively). Most learners were between 7 and 15 years of age (*n* = 182), 72 (35.5%) learners were 7–10 years of age, and 110 (54.2%) learners were 11–15 years of age. The participating parents’ age ranged from 19 to 59 years and older. Of these, 19 parents (26.8%) were aged 19–29 years, 28 (39.4%) were aged 30–39 years, and 24 (33.8%) were aged 40–≥60 years. The participating teachers were comparatively younger, with ages ranging from 19 to 39 years. Specifically, 9 teachers (50%) were aged 19–29 years, and 9 (50%) were aged 30–39 years. Nearly all participants self-identified as Black African, including 99% of learners, and 100% of both parents and teachers. Of the 71 participating parents, 58 had either some secondary education (*n* = 28, 39.4%) or had completed secondary education (*n* = 30, 42.3%). Most follow-up parents (*n* = 58, 81.7%) self-identified as engaged in ‘other’ forms of employment when asked about their employment status. A subset of follow-up participants living with HIV, including 3 learners (1.0%), 25 parents (35.2%), and 2 teachers (11.1%), while 2 learners (1.0%), 24 parents (33.8%), and 3 teachers (16.7%) reported current antiretroviral therapy (ART), 2 learners (1.0%), 5 parents (7.0%) and 4 teachers (22.2%) had reported having been on other medication. Only 4 learners self-reported comorbidities that may have qualified them for vaccination; only 1 learner reported being vaccinated. Among the follow-up population, one learner, 21 parents (29.6%) and 15 teachers (83.3%) reported a history of SARS-CoV-2 vaccination, as captured in the baseline survey; none reported receiving a primary or booster vaccination in the current follow-up survey (Table 1). Baseline characteristics of participants who completed follow-up were compared to those who did not (Supplementary Table 1). Among the 456 learners and 42 teachers enrolled in the baseline survey, similar characteristics were observed in those who were followed up (learners: *n* = 203 and teachers: *n* = 18) compared to those who were not (learners: *n* = 253 and teachers: *n* = 24) including gender, age, ethnicity, the grade of the learner, educational status of the teacher, employment status, comorbidities, concomitant meds, previous COVID-19 infection and vaccination status. Among parents, however, those who were followed up (*n* = 71) were significantly older (*p* = 0.03) and exhibited higher level of education (*p* = 0.03) compared to those who were not followed-up (*n* = 76) (Supplementary Table 1).

**Table 1:** Characteristics of the study participants by group at the follow-up survey.

Individual level characteristics	Learners in grades 1–7		Parents		Teachers	
	*n*	%	*n*	%	*n*	%
	203		71		18	
Grade						
Grade 1	21	10.3				
Grade 2	29	14.3				
Grade 3	27	13.3				
Grade 4	32	15.8				
Grade 5	27	13.3				
Grade 6	42	20.7				
Grade 7	25	12.3				
Grade categories						
Junior primary (grades 1–3)	77	37.9				
Senior primary (grades 4–7)	126	62.1				
Gender						
Male	95	46.8	9	12.7	2	11.1
Female	108	53.2	62	87.3	16	88.9
Other	–	–	–	–	–	–
Age						
<7	20	10.0	–	–	–	–
7–10	72	35.5	–	–	–	–
11–15	110	54.2	–	–	–	–
16–18	1	0.5	–	–	–	–
19–29	–	–	19	26.8	9	50.0
30–39	–	–	28	39.4	9	50.0
40–59	–	–	23	32.4	–	–
≥60	–	–	1	1.4	–	–
Ethnicity (race)						
Black African	201	99.0	71	100	18	100
Indian	–	–	–	–	–	–
Mixed race	2	1.0	–	–	–	–
White	–	–	–	–	–	–
Other	–	–	–	–	–	–
Education						
No formal education			3	4.2		
Junior primary			5	7.0		
Senior primary			1	1.4		
Some secondary			28	39.4		
Completed secondary			30	42.3		
Some university/technical			4	5.6		
Completed university/technical			–	–		
National certificate/trade						
Employment status						
Employed, part-time			–	–		
Employed, full-time			6	8.5		
Unemployed			7	9.9		
Other			58	81.7		
Comorbidities^*^						
HIV	3	1.0	25	35.2	2	11.1
TB (current)	0	0.0	0	0.0	1	5.6
TB (prior)	0	0.0	0	0.0	1	5.6
Diabetes mellitus	0	0.0	1	1.4	2	11.1
Other^#^	1	0.5	14	19.7	4	22.2
Concomitant meds						
ARVs/ART	2	1.0	24	33.8	3	16.7
Bactrim	0	0.0	0	0.0	0	0.0
TB meds	0	0.0	0	0.0	0	0.0
Other^#^	2	1.0	5	7.0	4	22.2
Previous COVID-19 infection						
Yes	0		2		1	
No	0					
Vaccination status						
Vaccinated	1	0.5	21	29.6	15	83.3
Unvaccinated	201	99.5	50	70.4	3	16.7

^*^Each comorbidity is expressed as a percentage of total participants, with multiple selections possible in responses. Other comorbidities include chronic kidney disease, chronic liver disease, heart disease, hypertension, asthma, chronic lung disease, rheumatological disease and obesity. ^#^Other concomitant medication includes steroids, anti-inflammatories, antihypertensives, hormonal treatment, antibiotics, aspirin/warfarin/heparin and anticoagulant.

### 3.2 SARS-CoV-2 seroprevalence overall and by participant groups at each survey

At baseline (*n* = 292), overall SARS-CoV-2 IgG seroprevalence was 73% (95% CI: 68–78%) unadjusted and 77.7% (95% CI: 70.6–81.1%) after adjustment for test sensitivity (Table 2). Whereas IgM was detected in 1.7% of participants (95% CI: 0.5–4.0%) unadjusted and 1.8% adjusted (95% CI: 0.8–4.1%), and 25% of participants tested negative (95% CI: 20–30%) unadjusted.

**Table 2:** Overall SARS-CoV-2 antibody seroprevalence and by group in the cross-sectional survey, unadjusted and adjusted for POC sensitivity of 96.2% and specificity of 100%.

Characteristic	Overall *N* = 292^1^ Unadjusted 95%Cl (Adjusted)	Learner *N* = 203^1^ Unadjusted 95%Cl (Adjusted)	Parent *N* = 71^1^ Unadjusted 95%Cl (Adjusted)	Teacher *N* = 18^1^ Unadjusted 95%Cl (Adjusted)	*p*-value^2^
Result					0.069
IgG positive	214 73% [68–78%] (77.7% [70.6–81.1%])	150 74% [67–80%] (76.8% [70.1–82.6])	47 66% [54–77%] (68.8% [56.8–79.1])	17 94% [73–100%] (98.8 [77.2–100]%)	
IgM positive	5 1.7% [0.5–4%] (1.8% [0.8–4.1%])	5 2.5% [1–6%] (2.7% [1.1–5.9])	0 0% [0–5%] (0% [0–5.3%])	0 0% [0–18%] (0% [0–18.3%])	
Negative	73 25% [20–30%]	48 24% [18–30%]	24 34% [23–46%]	1 5.6% [0.1–27%]	

^1^*n* (%). ^2^The *p*-value represents differences in the distribution of antibody results (IgG positive, IgM positive, and negative) across participant groups using Fisher’s exact test.^.^

Baseline IgG seroprevalence was higher among learners than parents (74% [95% CI: 67–80%] vs. 66% [95% CI: 54–77%] unadjusted; 76.8% (95% CI: 70.1–82.6%) vs. 68.8% (95% CI: 56.8–79.1%) adjusted), while teachers showed the highest prevalence (94% [95% CI: 73–100%] unadjusted; 98.8% [95% CI: 77.2–100%] adjusted). IgM seropositivity was detected only in learners (2.5%; 95% CI: 1–6% unadjusted; 2.7%; 95% CI: 1.1–5.9 adjusted). Twenty-four percent of learners (95% CI: 18–30%), 34% of parents (95% CI: 23–46%), and 5.6% of teachers (95% CI: 0.1–27%) tested seronegative (Table 2).

At follow-up (*n* = 292 matched), overall IgG seroprevalence increased to 89% (95% CI: 84.9–92.1%) unadjusted and 94.7% (95% CI: 91–97%) adjusted (Table 3). Learners had slightly higher IgG seroprevalence than parents (90% [95% CI: 84.7–93.1%] vs. 85% [95% CI: 74.3–91.1%]) unadjusted; (95.7% [95% CI: 92–98%] vs. 90.4% [95% CI: 81–95%]) adjusted. All the teachers who participated in the follow-up survey were IgG seropositive (95% CI: 82.4–100.0%). Ten percent of learners (95% CI: 6.9–15.3%) and 15% of parents (95% CI: 8.9–25.7%) tested seronegative (Table 3). The distribution of antibody results across participant groups did not differ significantly at baseline (Fisher’s exact test, *p* = 0.069) or at follow-up (*p* = 0.14).

**Table 3:** Overall SARS-CoV-2 antibody seroprevalence and by group in the follow-up survey, unadjusted and adjusted for POC sensitivity of 96.2%.

Characteristic	Overall *N* = 292^1^ Unadjusted 95%Cl (Adjusted)	Learner *N* = 203^1^ Unadjusted 95%Cl (Adjusted)	Parent *N* = 71^1^ Unadjusted 95%Cl (Adjusted)	Teacher *N* = 18^1^ Unadjusted 95%Cl (Adjusted)	*p*-value^2^
Result					0.14
IgG positive	260 89% [84.9–92.1%] (94.7% [91–97%])	182 90% [84.7–93.1%] (95.7% [92–98%])	60 85% [74.3–91.1%] (90.4% [81–95%])	18 100% [82.4–100.0%] (100% [82–100%])	
Negative	32 (11%) [7.9–15.1%]	21 (10%) [6.9–15.3%]	11 (15%) [8.9–25.7%]	0 (0%)	

^1^*n* (%). ^2^The *p*-value represents differences in the distribution of antibody results (IgG positive, IgM positive, and negative) across participant groups using Fisher’s exact test.

Follow-up IgG seroprevalence remained high across all participant groups irrespective of vaccination status (Supplementary Table 2). Among parents, 82.0% of unvaccinated participants and 90.5% of vaccinated participants were IgG seropositive. Among learners, 89.6% were IgG seropositive despite none having received a COVID-19 vaccine during the study period. All teachers were IgG seropositive at follow-up regardless of vaccination status; however, the teacher sample size was small (Supplementary Table 2).

### 3.3 Group-level longitudinal assessment of SARS-CoV-2 seroprevalence

**Figure 2:**
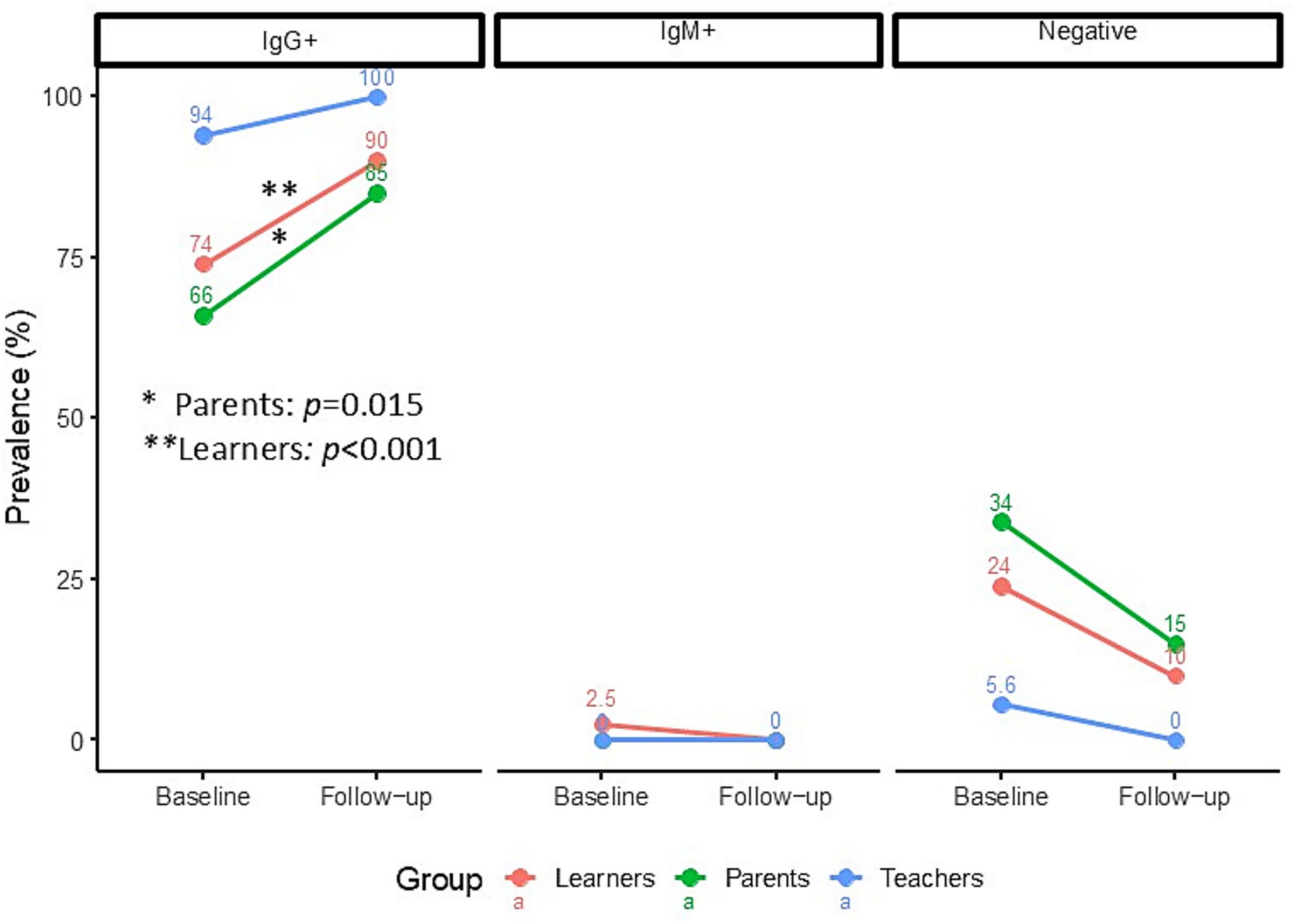
Change in SARS-CoV-2 seroprevalence (unadjusted) from baseline to the follow-up survey. *p*-values based on McNemar test at 5% level of significance.

Among the 292 participants with paired samples, IgG seroprevalence increased across all participant groups. Among learners, IgG seroprevalence increased significantly from 74 to 90% (*p* < 0.001), accompanied by a decline in IgM seropositivity from 2.5 to 0% and a decrease in seronegativity from 24 to 10%. Among parents, IgG seroprevalence rose from 66 to 85% (*p* = 0.015), with a corresponding decline in seronegativity from 34 to 15%. Among teachers, IgG seropositivity increased from 94% at baseline to 100% at follow-up, reflecting seroconversion of a single teacher who was seronegative at baseline (Figure 2).

### 3.4 Individual-level longitudinal assessment of SAR-CoV-2 seroprevalence

Among participants with paired SAR-CoV-2 antibody results (*n* = 287), IgG seroprevalence was high (Supplementary Table 2). Overall, 198 of 287 participants (68.9%) who were IgG positive at baseline remained seropositive at follow-up, while 16 (5.6%) seroreverted. Among the 73 participants who were seronegative at baseline, 57 (19.9%) seroconverted, while 16 (5.6%) remained seronegative. Similar patterns were observed across participant groups. Among learners, 70.7% of those who were IgG positive at baseline remained positive, while 18.7% of those initially seronegative seroconverted. Among parents, 57.7% remained IgG positive, and 26.8% of initially seronegative participants seroconverted. Almost all teachers who were IgG positive at baseline remained seropositive at follow-up, and the single teacher who was seronegative at baseline seroconverted (Supplementary Table 2). A McNemar’s test demonstrated a significant increase in IgG seropositivity between baseline and follow-up (*p* < 0.001), with substantially more participants seroconverting than seroreverting.

## 4 Discussion

### 4.1 Key findings

This study provides one of the first longitudinal assessments of SARS-CoV-2 seroprevalence in a South African school community, including learners in grades 1–7, their parents, and teachers who participated in the CoKiDSS survey between May–August 2023 and were followed up in October 2023. Overall, SARS-CoV-2 IgG seroprevalence was high throughout the study period, increasing from 78% in the initial survey to 95% at follow-up (adjusted for test sensitivity). Among learners, seroprevalence increased from 77 to 96%, and among parents from 69 to 90%, while teachers maintained consistently high seroprevalence (range of 99–100%). Despite low self-reported prior infection and limited vaccination coverage specifically among learners, IgG seroprevalence remained high across groups. These findings are consistent with national serosurveys from South Africa and the United Kingdom that have documented substantial increases in SARS-CoV-2 antibody prevalence following successive pandemic waves, particularly those driven by the Delta and Omicron variants (15, 41). However, in the absence of antigen-specific assays, quantitative antibody measurements, or virological surveillance limits attribution, the observed increase in IgG seroprevalence likely reflects a combination of ongoing SARS-CoV-2 exposures and antibody persistence. During the follow-up period, SARS-CoV-2 vaccination and immune-boosting programmes were not available for children and no vaccination campaigns targeted parents, suggesting that the increase in seropositivity may reflect a combination of factors, including new exposures, antibody persistence, immune boosting, or other unmeasured influences. Learners demonstrated the largest increase in SARS-CoV-2 IgG seroprevalence between the baseline and follow-up surveys, rising from 77 to 96%. Most learners reported no prior history of COVID-19 infection and had limited vaccination coverage during the study period. Hence, this pattern may be consistent with previously reported high rates of asymptomatic or mildly symptomatic SARS-CoV-2 infection in school-aged children. Similar patterns of increasing seroprevalence among children have been reported in several school-based studies conducted in Europe and North America (42–47). For example, the Ciao Corona longitudinal cohort in Switzerland documented an increase in seroprevalence from 5.6% in late 2020 to 31.1% in unvaccinated children by 2021, with further increases reported following subsequent pandemic waves (42). School-based studies in Italy, Germany, England, and the United States have likewise reported progressive increases in SARS-CoV-2 seroprevalence among children over time, particularly following the emergence of the Omicron variant (43–47). Evidence on SARS-CoV-2 seroprevalence in LMICs, including South Africa remains scarce, with just two notable studies to date, other than the CoKiDSS cross-sectional survey (32). A longitudinal, school-based cohort study implemented between December 2020 and April 2021 in hotspot zones and towns of the Oromia Region in Ethiopia reported that SARS-CoV-2 seroprevalence doubled within 4 months of reopening of schools from 25.7% at baseline to 46.3% in the second round using a Elecsys anti-SARS-CoV-2 anti-nucleocapsid assay (48). Similarly, a cross-sectional study conducted in public primary schools in Maputo City a Province in Mozambique between August 2022 and November 2022 described an 80.7% overall SARS-CoV-2 IgG or IgM seroprevalence (49). The high seroprevalence observed among learners in the CoKiDSS cohort is broadly consistent with these international observations.

Seroprevalence among parents also increased substantially between the baseline and follow-up surveys, rising from 69 to 90%. Vaccination coverage in this group was approximately 30%, based on parents’ self-reports of receiving a primary COVID-19 vaccination series and/or booster doses at baseline. As the assay used in this study detected antibodies against both spike and nucleocapsid antigens but did not distinguish between them, the observed IgG seropositivity among parents is likely due to a combination of prior infection, ongoing SARS-CoV-2 infection and vaccination. Consequently, the relative contribution of infection- and vaccine-induced antibodies cannot be determined. Similar patterns have been reported in population-based serosurveys in South Africa and other settings (15, 41), where high vaccination coverage among adults, together with widespread exposure during successive pandemic waves, has resulted in high overall seroprevalence.

Thus, the high seroprevalence observed among adults in this cohort may also reflect the combined effects of prior infection and vaccination, often referred to as *hybrid immunity*. Hybrid immunity occurs when individuals acquire immunity through both natural infection and vaccination and has been associated with broader and more durable immune responses compared with infection- or vaccine-induced immunity alone (50, 51). Although the assay used in this study does not allow differentiation between these immune sources, widespread exposure to SARS-CoV-2 during successive pandemic waves, together with vaccination among adults, may have contributed to the high antibody prevalence observed among parents and teachers.

Teachers consistently demonstrated the highest SARS-CoV-2 seroprevalence in both the baseline and follow-up surveys. This pattern may reflect several factors, including higher vaccination coverage among teachers, persistence of antibodies following both prior infection or vaccination, and potentially greater occupational exposure within school environments. Given the limitations of the qualitative serological assay used in this study, the relative contribution of infection- and vaccine-induced antibodies could not be determined. If higher occupational exposure contributed to the observed seroprevalence among teachers, this pattern is consistent with the prioritisation of educators in early COVID-19 vaccination strategies implemented in many settings. Similar findings were reported in the Ciao Corona study, where school staff, including teachers, demonstrated higher seroprevalence rates than learners, suggesting differences in exposure risk (52).

A substantial proportion of CoKiDSS participants demonstrated a persistent SARS-CoV-2 IgG antibody response over time. Three studies consistently show long-term persistence of SARS-CoV2 IgG antibodies following infection in children: the Italian longitudinal study observed that anti–Spike-RBD IgG lasted up to 10–12 months with children exhibiting significantly higher titers than adults (53); the Australian cohort detected IgG in asymptomatic or mildly symptomatic children 6 months post-infection (54); and a Spanish study reported 96% of pediatric patients retained anti SARS-CoV-2 IgG antibodies 1 year after infection (55). Similarly, across diverse adult cohorts, SARS-CoV-2 anti-spike IgG have demonstrated remarkable durability. In the large UK Biobank serology study of around 20,000 participants, 92% (95% CI: 90–93%) remained positive for spike-specific IgG 6 months post-infection, with gradual decline observed in nucleocapsid antibodies through 18 months (56). Additionally, a cohort of unvaccinated healthcare workers in Barcelona maintained combined IgG and IgA responses above 90% over 20.5 months, with 95.7% still IgG seropositive by November 2021 (57).^1^ Finally, a Lithuanian workplace outbreak followed over 13 months found that 83% (95% CI: 69–91%) of unvaccinated individuals remained IgG-positive, with antibody levels stable or gently declining between months 6 and 13 (58). Hence, both pediatric and adult populations demonstrate long-term persistence of SARS-CoV-2 IgG antibodies, especially against spike protein, offering insights into the durability of natural immunity across age groups. However, children maintain detectable SARS-CoV-2 IgG antibodies for about 6–12 months post-infection, but their levels decline more rapidly than in adults, who often preserve spike-specific IgG for 18–20 months with a gradual decrease over time. This contrast highlights age-related differences in the durability of antibody responses. CoKiDSS could not pinpoint the exact timing of SARS-CoV-2 infection, yet we observed that unvaccinated learners still had detectable IgG antibodies 3–4 months after the initial survey—evidence of sustained antibody responses.

Interestingly, a small subset of participants remained persistently negative for both SARS-CoV-2 IgG and IgM antibodies in both surveys. Firstly, the absence of detectable SARS-CoV-2 antibodies in these participants may be due to biological and technical factors, even in the context of prior exposure or infection. Individuals with asymptomatic or mild SARS-CoV-2 infections often generate weaker or short-lived humoral immune responses that lead to antibody titers that are below the assay detection threshold. In a study of asymptomatic SARS-CoV-2 infections in Wanzhou, China, researchers observed that during the acute phase, virus-specific IgG levels were much lower in asymptomatic individuals (median S/CO 3.4; IQR 1.6–10.7) than in symptomatic patients (median 20.5; IQR 5.8–38.2) (59). Further, in the early convalescent phase, 93% of asymptomatic cases showed a decline in IgG and 81% had reduced neutralizing antibodies, compared to 97 and 62%, respectively, in symptomatic patients. Secondly, the waning of antibodies over time may have contributed to the observed seronegativity in CoKiDSS learners and parents. The UK REACT-2 study reported a decline in antibody seropositivity in those who did not report a history of COVID-19, (−64.0, 95% CI −75.6, −52.3), compared to −22.3% (95% CI −27.0, −17.7) in those with SARS-CoV-2 infection confirmed on PCR (60). Additionally, the timing of SARS-CoV-2 antibody testing is crucial. Testing too early (<10 to 14 days post-infection) may miss seroconversion, while testing too late may yield false negatives due to waning antibodies (61). The test type and sensitivity are also important determinants. Serological assays differ in their ability to detect low antibody titres and in the specific antigens targeted (e.g., Spike vs. Neutralization protein). An assessment of rapid serological assays demonstrated significant variability and reported that there may be limitations in their use for COVID-19 diagnosis among the young (62). Finally, host factors like immunosuppression due to HIV infection (63) or corticosteroid use (64) could dampen the SARS-CoV-2 humoral immune response, leading to a seronegative test result despite confirmed prior COVID-19 infection. CoKiDSS was conducted in a setting with high HIV and TB prevalence, where immunosuppression could plausibly influence SARS-CoV-2 antibody responses, including seronegativity. However, we were unable to robustly assess the impact of HIV on serological outcomes due to reliance on self-reported HIV status and the absence of clinical or immunological data. This remains an important area for future research in similar high-burden settings. All five learners who were IgM seropositive in the baseline survey developed IgG antibodies by the follow-up survey, suggesting seroconversion over time. These findings suggest that learners likely experienced acute COVID-19 around the time of the CoKiDSS baseline survey and developed longer-term IgG responses by the follow-up survey.

Taken together, these findings indicate widespread SARS-CoV-2 antibody prevalence within this school community across learners, parents, and teachers during the study period. The high IgG seroprevalence observed across all groups may reflect cumulative exposure to SARS-CoV-2 during successive pandemic waves, together with contributions from vaccination among adults. Similar patterns of widespread seropositivity have been reported in national and international serosurveys conducted following the emergence of the Omicron variant, suggesting that a large proportion of populations had developed detectable antibodies by 2023. However, because the assay used in this study provides qualitative results and does not distinguish between antibodies arising from infection and those induced by vaccination, the relative contribution of these mechanisms cannot be determined. Nevertheless, these findings provide important insight into SARS-CoV-2 antibody dynamics within school communities in a South African setting characterised by high HIV and tuberculosis prevalence. Collectively, these findings underscore the value of school-based serological studies for understanding patterns of population-level exposure to emerging respiratory pathogens and for strengthening public health surveillance in community settings.

### 4.2 Strengths and limitations

This study provides one of the first longitudinal assessments of SARS-CoV-2 seroprevalence in a school-based community in South Africa, capturing antibody patterns among learners, their parents, and teachers over time. By following participants from an earlier cross-sectional survey, the study offers valuable insight into changes in serological status within an educational setting in a region characterised by a high burden of HIV and tuberculosis. The inclusion of both unadjusted and test-adjusted seroprevalence estimates further strengthens the robustness and interpretability of the findings. Additionally, the high follow-up rate enabled the detection of changes in serological status over time, including seroconversion and seroreversion.

Several limitations should be considered when interpreting the findings. The study design also did not allow determination of the precise timing of infection or the assessment of transmission dynamics within schools or households. Parents who participated in the follow-up survey were significantly older and more highly educated than those who did not follow up, introducing potential selection bias that may limit the representativeness of the sample and the generalizability of the findings.

SARS-CoV-2 antibodies were measured using a qualitative POC lateral flow assay that detects antibodies against both spike and nucleocapsid proteins. Consequently, antibody concentrations could not be quantified, and the assay could not distinguish between antibodies arising from natural infection and those induced by vaccination. Further, the observed increase in IgG seroprevalence over time may reflect several mechanisms, including antibody persistence following prior infection or vaccination, immune boosting after repeated exposures, or new infections, and these possibilities cannot be differentiated within the current study. The low IgM seroprevalence observed may reflect the transient nature of IgM response or assay limitations.

Future longitudinal studies incorporating quantitative serological assays, molecular surveillance, or viral sequencing would help contextualize serological findings and provide a more comprehensive understanding of SARS-CoV-2 transmission dynamics and immune responses within school communities.

### 4.3 Implications for research and public health

School-based serosurveillance may provide valuable insights into population-level exposure to pathogens such as SARS-CoV-2 within educational communities, which may either contribute to ongoing transmission or indicate the development of population immunity. As schools represent accessible and organized populations, they also offer practical settings for public health interventions. Such targeted surveillance can complement existing surveillance systems by identifying potential immunity gaps and informing public health planning. In settings with substantial occupational exposure, including schools, serological data may further enhance understanding of exposure patterns and transmission dynamics among both educators and learners.

## 5 Conclusion

In conclusion, this study provides the first longitudinal assessment of SARS-CoV-2 serology among primary school learners, their parents, and teachers in a South African school setting characterised by high HIV and tuberculosis prevalence. Overall, SARS-CoV-2 IgG seroprevalence remained high in the cohort and increased among learners and parents over the 3–4-month follow-up period, accompanied by declines in seronegativity and IgM positivity. The largest increases were observed among largely unvaccinated, predominantly asymptomatic learners, while teachers maintained consistently high IgG seropositivity. Though, in the absence of antigen-specific assays, quantitative antibody measurements, or virological surveillance, the observed increase in seroprevalence cannot be attributed to new SARS-CoV-2 infections alone and may also reflect antibody persistence or immune boosting following prior exposure. As SARS-CoV-2 vaccination and immune boosting were not available to children and no vaccination campaigns occurred among parents during the study period the findings most likely reflect new SARS-CoV-2 exposures together with antibody persistence among learners and parents. Overall, these findings should not be interpreted as evidence to guide vaccination policy but rather contribute to understanding SARS-CoV-2 antibody dynamics in school-aged populations in high-burden settings and highlight the potential role of school-based serosurveillance in monitoring population-level exposure to respiratory pathogens.

## Data Availability

Interested researchers should contact the corresponding author with a brief concept note describing the intended use and rationale for access. In accordance with the study’s data sharing policy, the dataset will be made available after completion and publication of the primary manuscripts by the Co-Principal Investigators. Additionally, approval will need to need to be obtained from the South African Medical Research Council Human Research Ethics Committee to access the de-identified dataset.

## Footnotes

1
https://pubmed.ncbi.nlm.nih.gov/36224590/?utm_source=chatgpt.com
