# Supplementary material for: Longitudinal SARS-CoV-2 seroprevalence among learners, parents, and teachers in a South African school community
Source: Front Public Health. 2026 Jun 19;14:1770703. doi: 10.3389/fpubh.2026.1770703 (PMC13328095; doi:10.3389/fpubh.2026.1770703)
Supplement: Supplementary file 1 [file Table_1.docx]

**Supplementary Table 1:** Characteristics of the study participants at baseline

|  | **Learners in grades 1-7**  **(n=456)** | | | **Parents**  **(n=147)** | | **Teachers**  **(n=42)** | | | |
| --- | --- | --- | --- | --- | --- | --- | --- | --- | --- |
| **Individual characteristics** | **Participants followed up**  **(n=203)** | **Participants not followed up**  **(n=253)** | **p-value** | **Participants followed up**  **(n=71)** | **Participants not followed up**  **(n=76)** | **p-value** | **Participants followed up**  **(n=18)** | **Participants not followed up**  **(n=24)** | **p-value** |
|  | n (%) | n (%) |  | n (%) | n (%) |  | n (%) | n (%) |  |
| **Grade**  Grade 1  Grade 2  Grade 3  Grade 4  Grade 5  Grade 6  Grade 7 | 21(10.3)  29(14.3)  27(13.3)  32(15.8)  27(13.3)  42(20.7)  25(12.3) | 36(14.2)  33(13.0)  37(14.6)  35(13.8)  25(10.0)  52(20.6)  35(13.8) | 0.778 |  |  |  |  |  |  |
| **Grade categories**  Junior Primary (grades 1-3)  Senior Primary (grades 4-7) | 77(37.9)  126(62.1) | 106(41.9)  147(58.1) | No p-value |  |  |  |  |  |  |
| **Gender (sex)**  Male  Female  Other | 95(46.8)  108(53.2)  - | 119(47.0)  134(53.0)  - | 1.000 | 9(12.7)  62(87.3)  - | 5(6.6)  71(93.4)  - | 0.265 | 2(11.1)  16(88.9)  - | 5(20.8)  19(79.2)  - | 0.679 |
| **Age**  < 7  7-10  11-15  16-18  19-29  30 - 39  40-59  ≥60 | 20(10.0)  72(35.5)  110(54.2)  1(0.5)  -  -  -  - | 26(10.2)  100(39.5)  125(49.4)  2(0.8)  -  -  -  - | 0.760 | -  -  -  19(26.8)  28(39.4)  23(32.4)  1(1.4) | -  -  -  10(13.2)  30(39.5)  28(37.0)  8(10.53) | 0.033 | -  -  -  9(50.0)  9(50.0)  -  -  - | 6(25.0)  7(29.2)  10(41.7)  1(4.2)  - | 0.064 |
| **Ethnicity**  Black African  Indian  Coloured  White  Other | 201(99.0)  -  2(1.0)  -  - | 251(99.2)  1(0.4)  0(0)  1(0.4)  - | 0.259 | 71(100)  -  -  -  - | 76(100) | No p-value | 18(100)  -  -  -  - | 24(100)  -  -  -  - | No p-value |
| **Education Status**  No formal education  Junior Primary  Senior Primary  Some Secondary  Completed Secondary  Some University/Technical  Completed University/Technical  National Certificate/Trade |  |  |  | 3(4.2)  5(7.0)  1(1.4)  28(39.4)  30(42.3)  4(5.6)  -  -  - | 1(1.3)  8(10.5)  8(10.5)  27(35.5)  29(38.2)  0(0.0)  2(2.6)  -  1(1.3) | 0.034 |  |  |  |
| **Is the parent currently employed?**  Employed, part-time  Employed, full-time  Unemployed  Other |  |  |  | 6(8.5)  7(9.9)  58(81.7)  0(0.0) | 12(15.8)  6(7.9)  57(75.0)  1(1.3) | 0.400 |  |  |  |
| **Comorbidities:**  HIV  TB (current)  TB (Prior)  Diabetes Mellitus  Other | 2(1.0)  0(0.0)  1(0.5)  0(0.0)  0(0.0) | 5(2.0)  1(0.4)  1(0.4)  0(0.0)  2(0.8) | No p-value | 11(15.5)  0(0.0)  3(0.4)  1(1.4)  8(11.3) | 25(33.0)  1(1.3)  4(5.3)  6(7.9)  8(10.5) | No p-value | 3(16.7)  0(0.0)  0(0.0)  2(11.1)  3(16.7) | 2(8.3)  0(0.0)  0(0.0)  1(4.0)  1(4.0) | No p-value |
| **Concomitant meds*:**  ARVs/ART  Bactrim  TB meds  Other^#^ | 1(0.5)  0(0.0)  0(0.0)  1(1.0) | 4(1.6)  0(0.0)  1(0.4)  0(0.0) | No p-value | 23(32.4)  0(0.0)  0(0.0)  5(7.0) | 26(34.2)  1(1.3)  0(0.0)  9(11.8) | No p-value | 1(5.6)  1(5.6)  0.(0.0)  4(22.2) | 2(8.3)  0(0.0)  0(0.0)  2(8.3) | No p-value |
| **Previous**  **COVID-19 infection**  Yes  No | 2(1.0)  201(99.0) | 1(0.4)  252(99.6) | No p-value | 4(5.7)  66(94.3) | 4(5.2)  73(94.8) | No p-value | 2(11.1)  16(88.9) | 0(0.00)  24(100.00) | No p-value |
| **Vaccination status**  Vaccinated  Unvaccinated | 1(0.5)  202(99.5) | 1(0.4)  252(99.6) | No p-value | 27(38.0)  44(62.0) | 33(43.4)  43(56.6) | No p-value | 15(83.3)  3(16.7) | 18(75.0)  6(25.0) | No p-value |

*Each comorbidity is expressed as a percentage of total participants, with multiple selections possible in responses. Other comorbidities include chronic kidney disease, chronic liver disease, heart disease, hypertension, asthma, chronic lung disease, rheumatological disease and obesity

^#^Other concomitant medication includes steroids, anti-inflammatories, antihypertensives, hormonal treatment, antibiotics, aspirin/warfarin/heparin and anticoagulant

^$^P-values were not calculated for variables with no variability or sparse cell counts where statistical comparison was not appropriate.

**Supplementary Table 2. SARS-CoV-2 IgG seroprevalence at follow-up by COVID-19 vaccination status**

| **Participant group** | **Vaccinated** | **Negative n (%)** | **IgG positive n (%)** | **Total** |
| --- | --- | --- | --- | --- |
| **Parents (n = 71)** | No | 9 (18.0) | 41 (82.0) | 50 |
|  | Yes | 2 (9.5) | 19 (90.5) | 21 |
| **Learners (n = 202)** | No | 21 (10.4) | 181 (89.6) | 202 |
| **Teachers (n = 18)** | No | 0 (0) | 3 (100) | 3 |
|  | Yes | 0 (0) | 15 (100) | 15 |

**Supplementary Table 3. Changes in SARS-CoV-2 IgG serostatus between baseline and follow-up**

| **Serological transition** | **Overall (N=287*)** | **Learners (n=198*)** | **Parents (n=71)** | **Teachers (n=18)** |
| --- | --- | --- | --- | --- |
| **IgG persistence (positive → positive)** | 198 (68.9%) | 140 (70.7%) | 41 (57.7%) | 17 (94.4%) |
| **Seroreversion (positive → negative)** | 16 (5.6%) | 10 (5.1%) | 6 (8.5%) | 0 |
| **Seroconversion (negative → positive)** | 57 (19.9%) | 37 (18.7%) | 19 (26.8%) | 1 (5.6%) |
| **Remained seronegative (negative → negative)** | 16 (5.6%) | 11 (5.5%) | 5 (7.0%) | 0 |

*Participants with IgM-positive baseline results (n = 5) were excluded from persistence/change calculations.
